# ZNFX1: a multifunctional modulator of the innate immune response

**DOI:** 10.3389/fimmu.2025.1564628

**Published:** 2025-03-18

**Authors:** Li Yi Cheng, Roy Parker

**Affiliations:** ^1^ Department of Biochemistry, University of Colorado Boulder, Boulder, CO, United States; ^2^ Howard Hughes Medical Institute, University of Colorado Boulder, Boulder, CO, United States

**Keywords:** ZNFX1, innate immunity, chronic inflammation, antiviral response, RNA helicase, zinc finger protein, NLRP3 inflammasome

## Abstract

Recent research has identified ZNFX1 as a critical modulator of the innate immune response. Individuals with loss of function mutations in ZNFX1 have chronic inflammation and increased susceptibility to various pathogens. Several potential functions of ZNFX1 have been proposed, including binding double-stranded RNA to activate antiviral innate immunity, inhibiting the NLRP3 inflammasome, and regulating the stability of host mRNAs. Notably, homologs of ZNFX1 are implicated in innate immunity across a wide range of species, including contributing to transgenerational epigenetic inheritance of small RNA-based defense in *C. elegans*. In this review, we discuss the significance of ZNFX1 and explore the potential underlying mechanisms that govern its diverse functions.

## 1 ZNFX1 is a regulator of innate immunity

Humans encounter millions of infectious agents every day, and the innate immune response forms the first line of host defense against a variety of pathogens in our surrounding environment. The innate immune responses does not target specific infectious agents, rather it uses an arsenal of cell surface, endosomal and cytoplasmic receptors to recognize conserved features on pathogens that are generally absent in a healthy host. For example, toll-like receptors recognize pathogen-associated molecular patterns including lipopolysaccharide, peptidoglycan and bacterial flagella, while RNA helicases such as MDA5 and LGP2 detect cytoplasmic double-stranded RNAs. Together, these conserved sensors activate downstream interferon signaling to mount a more robust immune response to fight infection, to warn neighboring uninfected cells, and to activate the adaptive immune response. However, hyperactivation of host immunity is undesirable, especially when the initial infection has been contained. An overactive innate immune system can lead to persistent inflammation and tissue damage that can result in high morbidity and mortality. Proper regulation of the innate immune responses is therefore critical to balance inflammatory signals and maintain normal cellular homeostasis.

In recent years, an understudied protein known as NFX-1 type zinc finger-containing protein 1 (ZNFX1) has been implicated as an important immunomodulator of the innate immune system. ZNFX1 deficiency is an autosomal recessive disorder. To date, all patients have carried either homozygous or *trans* compound heterozygous pathogenic variants of the *ZNFX1* gene, leading to a loss of ZNFX1 expression (1). Individuals with ZNFX1 deficiency suffer from altered immune regulation. Such patients have predisposition to viral infections and severe multisystem inflammatory diseases including cytopenia, hepatitis, seizures, renal and lung disease (1–3). These disease manifestations often start early in childhood and exhibit a high mortality rate (1). Recently, ZNFX1 deficiency has also been associated with increased susceptibility to mycobacterial diseases (4, 5).

Mice with Znfx1 deficiency also have aberrant immunoregulation phenotypes. Specifically, in *Znfx1^-/-^* C57BL/6J mice infected with Vesicular Stomatitis Virus (VSV) or (*M. tuberculosis*), pathogen load was elevated by at least 2-fold compared to WT mice (5, 6). In addition, 80% of *Znfx1^-/-^* mice infected with VSV demonstrated conjunctivitis one day post infection, and their lungs showed more infiltration by inflammatory cells compared to controls (6). Most strikingly, *Znfx1^-/-^* mice showed a significant reduction in overall survival after VSV infection (6) and LPS treatment (7).

Consistent with ZNFX1 contributing to proper regulation of immune responses, ZNFX1 is induced by infections in many species. ZNFX1 expression was upregulated in SARS-CoV-2-infected cynomolgus macaque lungs (8). Chickens treated with double-stranded RNA (dsRNA) poly(I:C) had increased ZNFX1 expression (9). In fish, ZNFX1 transcripts were upregulated in Atlantic cod brains with nodavirus (10) and in grouper fish infected with nervous necrosis virus (11). Intriguingly, bioinformatic analyses identified that the promoter region of *ZNFX1* contained binding motifs for proteins involved in Type I interferon (IFN) signaling pathway, such as STAT1/2, IRF1 and IRF9 (6), reinforcing its function in antiviral immunity.

ZNFX1 can also be induced by environmental stress. In salmon after smoltification (12) and in oysters responding to hypoosmotic stress (13), ZNFX1 is shown to be upregulated.

ZNFX1 might be involved in different aspects of immunoregulation throughout the stages of an infection. In the early stages of an infection, ZNFX1 could serve to promote viral clearance. ZNFX1 patient-derived monocytes were shown to be less efficient in clearing virus compared to controls after being subjected to poly(I:C) pre-stimulation and infection with VSV *in vitro (* 1). In addition, transcriptomics of 3044 COVID-19 cases revealed that ZNFX1 expression level had the strongest negative correlation with SARS-CoV-2 viral load (14). At later stages of an infection or when an infection is cleared, ZNFX1 could function to balance inflammatory signals and prevent persistent inflammation. In the peripheral blood of ZNFX1-deficient patients who do not have any known ongoing infections, many interferon-stimulated genes (ISGs) involved in antiviral and PAMP sensing such as OAS1/2, IFIT2/3 and IRF7 were detected at a higher level compared to healthy controls (1). This suggests that either an initial infection is never fully cleared in the absence of ZNFX1 or ZNFX1 deficiency causes dysregulation in the immune response that prevents cells from returning to normal homeostasis after the infection is cleared.

Taken together, the induction of ZNFX1 in response to pathogens, and the loss of proper immunoregulation in ZNFX1-deficient humans and animals, argue that ZNFX1 plays an important function in innate immunity control.

### 1.1 ZNFX1 and its domains are evolutionarily conserved

Several observations argue that ZNFX1 is a conserved protein constantly under purifying selection. First, sequences with similar protein domain organization to the human *ZNFX1* have been found in both vertebrates and invertebrates. Second, ZNFX1 exhibits limited sequence diversity within vertebrates (15). Third, population genetics analyses in humans show that there are no healthy homozygotes found for predicted loss-of-function (LoF) variants of ZNFX1 (gnomAD v2.1.1). Finally, ZNFX1 also has a low Loss-of-function Observed/Expected Upper-bound Fraction (LOEUF) score of 0.25 (gnomAD v2.1.1). Low genetic constraint scores indicate a strong correlation with gene essentiality, with haploinsufficient genes having a natural cut-off at 0.2 (16). Taken together, these observations demonstrate that ZNFX1 is constantly under purifying selection and suggests that it is an evolutionary conserved protein with important cellular function.

**Figure 1:**
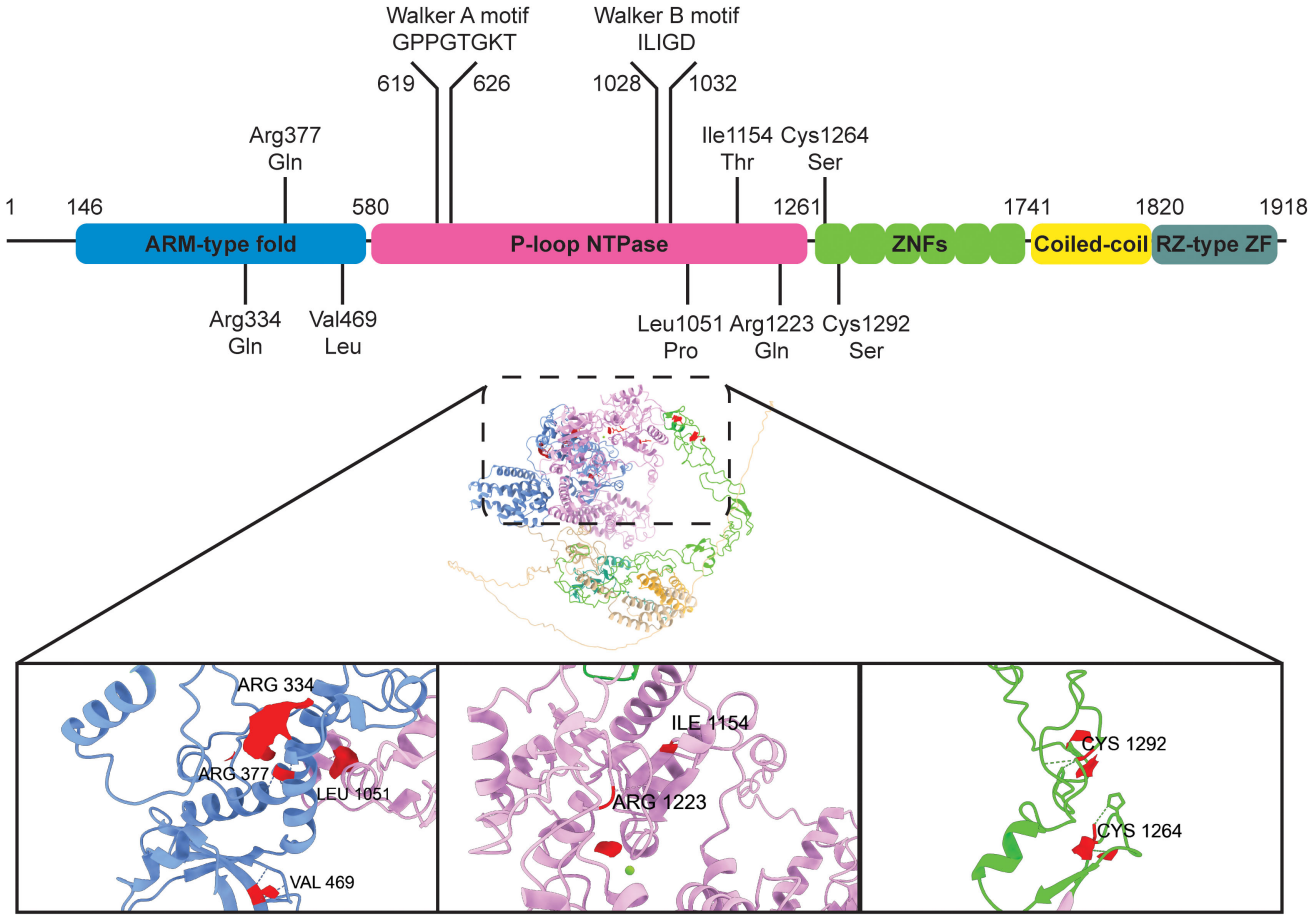
ZNFX1 protein domains and predicted structure. Diagram of ZNFX1 protein domains, with pathogenic missense mutations highlighted. ZNFX1 structure predicted using AlphaFold3 (37) is shown (predicted template modelling score = 0.54), protein domains are colored corresponding to that in the sequence. Amino acid residues involved in pathogenic missense mutations are displayed in red. Green ball = Mg^2+^ ion.

The structural features of ZNFX1 offer a hint into its function in immune regulation (**Figure 1**).

In humans, ZNFX1 encodes a 1918 amino acid protein. ZNFX1 is a superfamily (SF) 1 RNA helicase containing an Armadillo-type fold (ARM) domain, a P-loop NTPase domain, six NFX-1 type zinc fingers, a coiled-coil region and a C-terminus RZ-type zinc finger.

Zinc fingers are one of the most abundant protein domains. Given the wide variety of zinc finger types, it is not surprising that zinc finger proteins are implicated in an array of molecular functions that range from transcriptional regulation to protein degradation. Yet, both the NFX-1 type and RZ-type zinc fingers in ZNFX1 are non-classical protein domains, and their biological properties are largely unexplored. The NFX-1 type zinc finger has only been shown to bind DNA in the transcriptional regulator, NFX1 where it interacts with the X-box motif in promoters (17). On the other hand, the name “RZ-type zinc finger” is derived from RNF213 and ZNFX1, the only two known proteins that share sequence similarity to this peptide motif. In RNF213, an E3 ubiquitin ligase, the RZ-type zinc finger is found to be essential for both autoubiquitylation and ubiquitylation of lipopolysaccharide during Salmonella infection (18). Therefore, the zinc finger domains in ZNFX1 can possibly interact with a diverse range of molecular substrates, from nucleic acids to proteins.

ZNFX1 also contains the seven motifs found in SF1 and SF2 DNA/RNA helicases. In particular, the core helicase domain of ZNFX1 exhibits a typical Rossmann fold with the highly conserved Walker A (GxxGxGK(T/S)) and Walker B (hhhhD) motifs. Structural modelling of ZNFX1 from nine different species showed good conservation of the ARM-type fold and P-loop NTPase domain, suggesting that the ARM-type fold could be a unique feature of ZNFX1 that distinguishes it from other SF1 RNA helicases and indicative of its unique cellular function (15). Additionally, variable domains in between the RecA domains of the P-loop helicase could also facilitate ZNFX1 binding to different interacting partners. These features suggest that ZNFX1 binds nucleic acids in an ATP-dependent manner. Since interactions with binding partners often modulate the nucleic acid binding and/or ATP hydrolysis rates of SF1/SF2 family members, one anticipates that binding of ZNFX1 to one or more types of nucleic acids will be influenced by additional protein interactions.

## 2 Subcellular localization of ZNFX1

**Figure 2:**
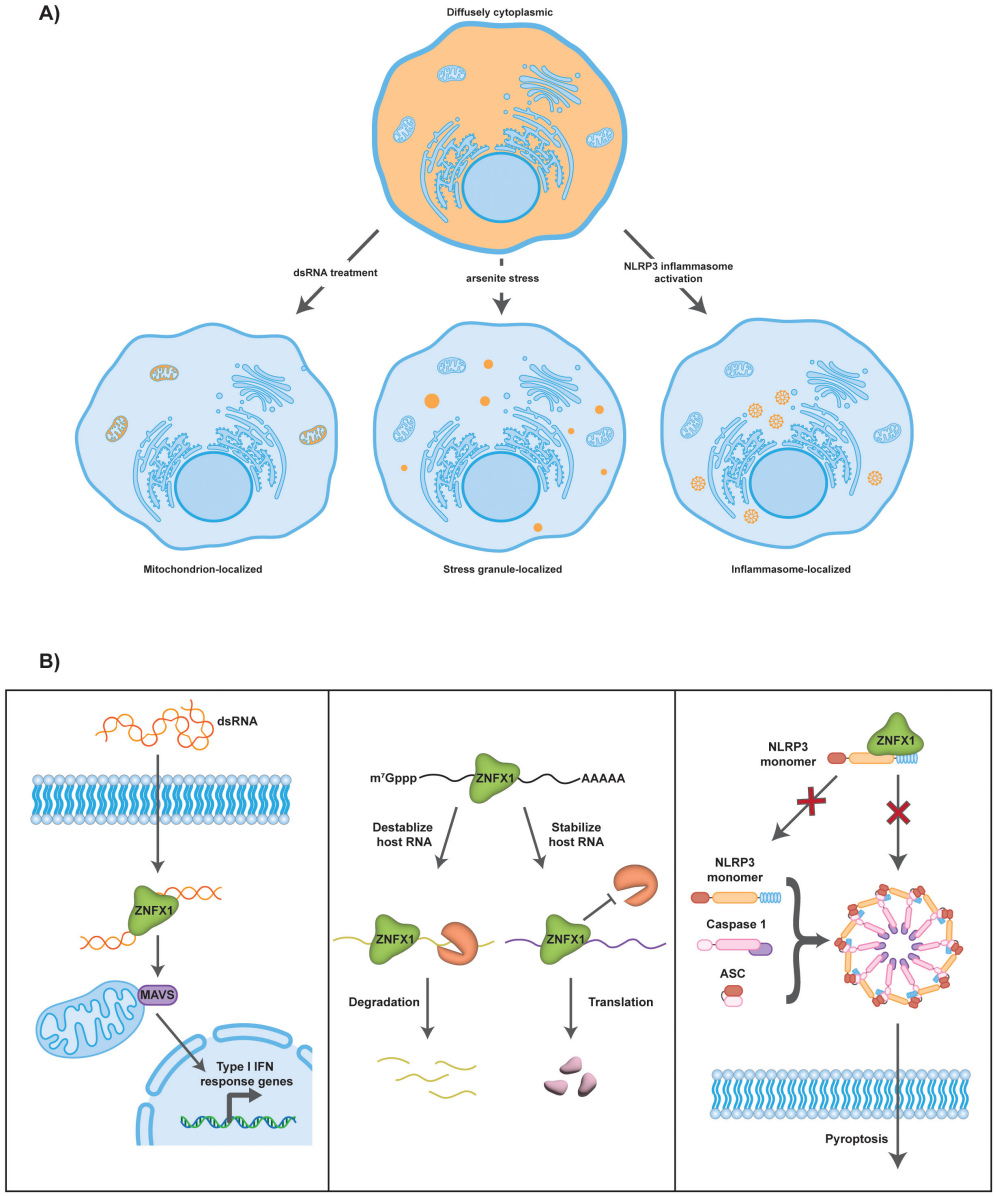
Subcellular localization and proposed molecular mechanisms of ZNFX1. **(A)** Under normal cellular conditions, ZNFX1 is diffusely localized throughout the cytoplasm. When cells are subjected to varying conditions, subcellular localization of ZNFX1 becomes altered. During dsRNA treatment, ZNFX1 localizes to the mitochondria through its interaction with MAVS. During arsenite treatment, ZNFX1 enriches in stress granules. During inflammasome activation, ZNFX1 localizes to NLRP3 inflammasomes and ASC specks. **(B)** ZNFX1 is proposed to function via three molecular mechanisms. (Left) ZNFX1 acts as a dsRNA sensor. It recognizes and binds intracellular viral dsRNA. Through its interaction with MAVS at the outer mitochondrial membrane, the downstream Type I interferon signaling pathway becomes activated. (Middle) ZNFX1 regulates host mRNA stability. It can either cause mRNA degradation as shown for several ISG mRNAs or promote mRNA stability in the case of *Prkaa2* mRNA. (Right) ZNFX1 inhibits NLRP3 inflammasome activation. Binding of ZNFX1 to NLRP3 could prevent NLRP3 activation and formation of mature NLRP3 inflammasomes with ASC and Caspase 1, thereby inhibiting pyroptosis.

ZNFX1 is a cytosolic protein, but has been described to localize to different subcytoplasmic locations under different contexts (**Figure 2A**) (4–7). During VSV infection, ZNFX1 is proposed to bind to mitochondrial antiviral-signaling protein (MAVS) and localize to the outer mitochondria membrane (6). Recently, new evidence suggested that ZNFX1 binds to NACHT, LRR and PYD domains-containing protein 3 (NLRP3) monomers (7). More specifically, in resting cells, ZNFX1 is diffusely localized throughout the cytoplasm to prevent NLRP3 oligomerization and keep it in an inactive state (7). During NLRP3 activation, NLRP3 brings ZNFX1 to ASC specks to be cleaved by caspase 1 (7). The connection to NLRP3 is of particular interest since several ZNFX1 patient mutations abolished NLRP3 interaction and led to increased NLRP3 puncta under resting conditions (7).

ZNFX1 has also been observed to accumulate in stress granules (4), which are assemblies of untranslating messenger ribonucleoproteins (RNPs) that form when translation initiation is inhibited (19). In a recently published time-resolved mRNA interactome study, ZNFX1 was also found in the same cluster as many known stress granule proteins (20). Interestingly, in the absence of any additional stressor, overexpression of ZNFX1 also led to stress granule formation (4). It is thought that protein overexpression can generally lead to stress granule formation either by repressing translation initiation and/or by serving as a scaffold to promote RNP self-assembly, although it is not yet understood as to how ZNFX1 works in this regard.

## 3 Molecular mechanisms of ZNFX1 function

Three mechanisms have been proposed for how ZNFX1 regulates the immune response (**Figure 2B**), and these are not mutually exclusive.

### 3.1 ZNFX1 as a sensor of dsRNA

In the first model, ZNFX1 is proposed to function as a dsRNA sensor that activates MAVS-dependent immunosignaling (6). The key observation is that ZNFX1 in cell lysates binds to exogenous poly(I:C) and this binding is only competed away with high molecular weight poly(I:C), but not with low molecular weight poly(I:C), poly(dA:dT) or 5’ ppp dsRNA (6). Moreover, when pulling down ZNFX1 in VSV-infected 293T cells, VSV viral RNA with an average length of 6 kilobases co-precipitated with ZNFX1, although whether this was ssRNA or dsRNA was not determined. By pulling down on different ZNFX1 domain mutants transiently transfected into HEK293T cells, the authors showed that 1) ZNFX1-poly(I:C) interaction required both the ARM-domain and the P-loop NTPase domain and 2) ZNFX1’s ARM domain associated with the transmembrane domain of MAVS. Together, these observations raise the possibility that ZNFX1 could be another dsRNA sensor, alongside LGP2 and MDA5, to detect intracellular viral RNAs and activate downstream interferon signaling through MAVS.

### 3.2 ZNFX1 as a regulator of mRNA stability

A second proposed function of ZNFX1 is to bind and modulate stability of host mRNAs. Evidence that ZNFX1 could regulate host mRNA decay rates comes from two independent studies.

First, ZNFX1 has been suggested to suppress mycobacterial growth by stabilizing *Prkaa2* mRNA transcripts via its C-terminal zinc finger regions (5). This research started with the observation that Znfx1 was upregulated following *M. tuberculosis* infection in mouse bone marrow derived macrophages (BMDMs) (5). Loss of Znfx1 led to compromised immune responses in *M. tuberculosis*-infected macrophages, as well as tissue injury and increased bacterial burden in *Znfx1^-/-^* mice post *M. tuberculosis* infection. However, no difference was observed in ISG expression between *Znfx1^-/-^* and WT cells after LPS and VSV treatment in BMDMs, suggesting Znfx1 did not alter the interferon response in this context. Notably, the human ortholog of ZNFX1 has also been associated with Mendelian susceptibility to mycobacterial diseases (4). Patients from two unrelated families who carry homozygous *ZNFX1* variants have been reported to exhibit mycobacterial disease alongside intermittent monocytosis (4), which is a condition characterized by temporary increases in monocytes and likely to be indicative of infection.

During mycobacterial infections, ZNFX1 was suggested to regulate the decay rate of *Prkaa2* mRNA for three reasons (5). First, *Prkaa2* mRNA levels increased during *M. tuberculosis* infection but remained at basal levels in *Znfx1^-/-^* BMDMs. Second, in *Znfx1^-/-^* BMDMs, *Prkaa2* mRNA decayed faster than in WT when RNA polymerase II was inhibited with 5,6-dichlorobenzimidazole. Finally, *Prkaa2* mRNA showed significant enrichment in an ZNFX1 immunoprecipitation assay in a manner dependent on the zinc finger region of ZNFX1. These observations gave rise to the model that ZNFX1 can specifically bind host mRNAs and increase their stability.

Conversely, another study proposed that ZNFX1 could also promote the degradation of host mRNAs. The primary observation was that ISG mRNAs including *IFIT1/2* and *OAS1/2* showed increased stability in ZNFX1-deficient patient fibroblasts compared to controls when transcription was inhibited 18 hours post poly(dA:dT) stimulation (1). However, whether ZNFX1 directly binds to these ISG mRNAs to regulate their stability is not known. In this case, it suggests that ZNFX1 could be involved in balancing host immune responses by degrading pro-inflammatory transcripts to prevent immune hyperactivation.

This is reminiscent of an early discovery regarding the human NFX1 protein which features several NFX1-type zinc finger regions similar to those found in ZNFX1. NFX1 is believed to function as a transcription repressor of class II major histocompatibility complex (MHC) genes using its cysteine-rich zinc finger domains (17). Under normal conditions, its cellular expression is low but gets drastically upregulated in the later stages of IFNγ treatment (17). Since the upregulation of NFX1 coincides with the inhibition of class II MHC genes, NFX1 is thus thought to control the immune response. What is more intriguing is that NFX1 has been shown to bind an oncoprotein, human papillomavirus (HPV) type 16 E6 (16E6) (21). Furthermore, a splice variant known as NFX1-123 is proposed to aid in immune evasion of HPVs and cause cervical carcinogenesis. Specifically, overexpression of NFX1-123 in human foreskin keratinocytes suppressed the expression of pro-inflammatory cytokines and ISGs, including CXCL1, TNF, OAS1/2 by altering the subcellular localization of immune signaling proteins such as TRAF6 and TAB2 (22). Given the similarity in NFX1-type zinc fingers between NFX1 and ZNFX1, it is plausible that zinc finger regions of ZNFX1 could mediate host RNA expression following an infection.

### 3.3 ZNFX1 as an inhibitor of the NLRP3 inflammasome

ZNFX1 has recently been proposed to function as an inhibitor of the NLRP3 inflammasome (7). The primary observation was that activation of NLRP3 by nigericin, whose action mimics potassium efflux by equilibrating potassium ions across membranes, in ZNFX1-deficient macrophages resulted in a significant increase in cleaved caspase-1 and IL-1β levels. Additionally, in the absence of ZNFX1, there was enhanced translocation of NLRP3 into TGN46+ vesicles, and increased formation of ASC specks, both of which are consistent with overactivation of the NLRP3 inflammasome. Reinstating wildtype, but not alleles with patient-derived mutations, ZNFX1 protein successfully rescued these phenotypes. Moreover, injection of *Znfx1^-/-^* mice with lipopolysaccharide led to activation of NLRP3 inflammasome, elevated levels of IL-1β and higher mortality rate compared to wildtype mice. These phenotypes argue that the failure to suppress NLRP3 activation is related to the clinical presentation of patients with LOF alleles of ZNFX1. It is also noteworthy that the effect of ZNFX1 on survival in both mouse models and human patients could indicate a more complex role for ZNFX1 in regulating the innate immune response.

Mechanistically, it has been suggested that ZNFX1 represses NLRP3 through direct binding of ZNFX1 to NLRP3 to prevent NLRP3 oligomerization, thereby suppressing inflammasome activation. Using overexpressed domain truncations of ZNFX1 and NLRP3, the ARM domain and P-loop NTPase domain of ZNFX1, along with the NACHT and LRR domains of NLRP3 were suggested to be involved in the ZNFX1-NLRP3 interaction (7). Collectively, these observations indicate that ZNFX1 might be involved in suppressing the NLRP3 inflammasome.

However, since the precise mechanisms governing NLRP3 activation are still under active investigation, the exact way in which ZNFX1 interacts with NLRP3 may be subject to debate. For example, recent characterization of NLRP3 using cryo-electron microscopy approaches have demonstrated an additional regulatory step during NLRP3 activation (23, 24). In both murine and human cells, NLRP3 oligomerizes into an inactive double-ring cage structure that subsequently undergoes conformational change to form the active inflammasome in response to a priming signal (23, 24). Therefore, apart from binding to monomeric NLRP3, there exists a possibility where ZNFX1 could also interact with the NLRP3 double-ring structure to prohibit specific conformational changes and prevent NLRP3 activation.

An interesting comparison can be made with other DExD/H-box proteins that are believed to interact with NLRP3 and influence its activity. Several studies have demonstrated that the DEAD-box helicase DDX3X mediates NLRP3 activation (25, 26). During a viral infection, it is thought that DDX3X coordinates the crosstalk between stress granule formation and NLRP3 assembly. DDX3X normally participates in stress granule assembly during an infection to facilitate translation shutoff and activate interferon responses (26, 27). However, when a virus inhibits stress granule formation, DDX3X can instead promote NLRP3 activation and cause pyroptosis as another antiviral strategy (25, 26). Similar findings have also been reported with DHX33 and DDX19A, both of which have been implicated as cytosolic RNA sensors that bind viral RNA and initiate NLRP3 inflammasome activation (28, 29). Therefore, cells possess an arsenal of RNA helicases where each potentially exhibits specificity towards different RNA species. These RNA helicases can either work together or engage in competing roles to activate or inhibit the inflammasome. This diversity therefore allows the cell to regulate the NLRP3 inflammasome through the coordinate action of these RNA sensors.

A role of ZNFX1 in regulating the NLRP3 inflammasome could explain why ZNFX1 is upregulated in chronic illnesses that are associated with persistent inflammation. For example, ileum transcriptomics of individuals afflicted with asthma showed a more than 2-fold increase in ZNFX1 expression (30). ZNFX1 may thus act to subdue an overzealous immune system during chronic inflammation.

### 3.4 ZNFX1 function in *C. elegans*

In the nematode, *Caenorhabditis elegans*, the ZNFX1 ortholog also functions in host defense, but through modulating transgenerational epigenetic inheritance. In *C. elegans*, RNA-mediated interference uses short interfering RNAs to repress foreign RNA during infection, as well as endogenous genes during development (31). This system can also function across generations by maintaining a pool of heritable small RNA species in the progenies (32). ZNFX1 is believed to be required for the maintenance of heritable small RNAs in two manners. First, based on a yeast-two-hybrid experiment, the Tudor/LOTUS domain of LOTR1 binds directly to the N-terminal of ZNFX1 to stabilize the small RNA amplifying machinery at the 3’ end of target mRNAs (33). In addition, ZNFX1 also works with WAGO proteins to achieve maximum silencing in subsequent generations not only by amplifying a target group of small RNAs, but more importantly by maintaining of a pool of transcripts with poly(UG) tails in RNA-rich condensates of *C. elegans* germ cells called the nuage to be used as templates for small RNA amplification to achieve inheritance (34, 35).

While it is unclear if ZNFX1 directly binds RNAs and/or recognizes specific RNA sequence motifs to maintain epigenetic inheritance, the RNA binding function of ZNFX1 was shown to be essential for this role (34, 36). In particular, the helicase-null ZNFX1 failed to co-precipitate with mRNAs targeted for heritable gene silencing (36) and had defects in maintaining transgene silencing in subsequent generations (34). Therefore, in this context, the involvement of nematode ZNFX1 in transgenerational epigenetic inheritance could be reminiscent of (or analogous to) its role in host immunity in vertebrate organisms. Understanding how the evolution of these apparently different functions of a conserved protein will require more effort to elucidate the detailed molecular functions of ZNFX1 in both *C. elegans* and humans.

## 4 Conclusions and perspectives

Current work shows that ZNFX1 is a crucial multifunctional immunomodulator and most likely an interferon stimulated gene, with significant biological importance in the innate immune system across many animal species. It is subject to strong negative selection pressure and is of clinical importance as individuals with homozygous variants of ZNFX1 are susceptible to multisystem inflammatory diseases and face a heightened risk of childhood mortality. Given this significance, it will be essential to understand the specific molecular function(s) of ZNFX1.

Based on cell biological assays, ZNFX1 is proposed to function via three mechanisms, including sensing double-stranded RNA, modulating mRNA decay, and suppressing NLRP3 inflammasome activation. These functions may be synergistic in nature, with ZNFX1 potentially adopting different roles at different times depending on the immune context. Alternatively, some of these phenotypes could be secondary and downstream of initial alterations. This could be approached with biochemical assays to reproduce ZNFX1 functions and interactions *in vitro*. A critical aspect will be to connect specific phenotypes of ZNFX1 to function using pathogenic mutations that drive human disease, as has been initially done with ZNFX1 interactions with NLRP3.

In addition, the exact biological functions of the P-loop NTPase domain and zinc fingers of ZNFX1 remain largely unexplored. One can imagine a scenario in which ZNFX1 initially recognizes and binds to nucleic acids through its helicase core in an ATP-dependent manner. Following ATP hydrolysis, ZNFX1 not only resolves secondary structures in the target nucleic acid but, more importantly, undergoes a conformational change that enables it to engage in protein-protein interactions using the ARM domain or zinc fingers, thereby conferring its role in immunoregulation. Moving forward, it will be important to decipher the protein structure of ZNFX1, as well as when it is complexed with RNAs and/or proteins, to elucidate the precise functions of ZNFX1 and its domains.

Understanding the mechanisms of synergy and interplay between the diverse functions of ZNFX1 could therefore provide valuable knowledge on how the innate immune system coordinates an effective inflammatory response against pathogens and mitigate excessive immune response.

## References

[1] Vavassori S Chou J Faletti LE Haunerdinger V Opitz L Joset P. Multisystem inflammation and susceptibility to viral infections in human ZNFX1 deficiency. J Allergy Clin Immunol. (2021) 148:381–93. doi: 10.1016/j.jaci.2021.03.045 PMC8569286 33872655

[2] Alawbathani S Westenberger A Ordonez-Herrera N Al-Hilali M Al Hebby H Alabbas F. Biallelic ZNFX1 variants are associated with a spectrum of immuno-hematological abnormalities. Clin Genet. (2022) 101:247–54. doi: 10.1111/cge.14081 34708404

[3] Al-Saud B Alshareef T Al-Alwan M Alazami AM. ZNFX1 deficiency in a child with interstitial pneumonitis and peripheral monocytosis. J Clin Immunol. (2023) 43(7):1529–32. doi: 10.1007/s10875-023-01529-0 PMC10250176 37291413

[4] Le Voyer T Neehus A-L Yang R Ogishi M Rosain J Alroqi F. Inherited deficiency of stress granule ZNFX1 in patients with monocytosis and mycobacterial disease. Proc Natl Acad Sci. (2021) 118:e2102804118. doi: 10.1073/pnas.2102804118 33876776 PMC8053974

[5] Liu H Han Z Chen L Zhang J Zhang Z Chen Y. ZNFX1 promotes AMPK-mediated autophagy against *Mycobacterium tuberculosis* by stabilizing *Prkaa2* mRNA. JCI Insight. (2024) 9. doi: 10.1172/jci.insight.171850 PMC10906457 38016036

[6] Wang Y Yuan S Jia X Ge Y Ling T Nie M. Mitochondria-localised ZNFX1 functions as a dsRNA sensor to initiate antiviral responses through MAVS. Nat Cell Biol. (2019) 21:1346–56. doi: 10.1038/s41556-019-0416-0 31685995

[7] Huang J Wang Y Jia X Zhao C Zhang M Bao M. The human disease-associated gene ZNFX1 controls inflammation through inhibition of the NLRP3 inflammasome. EMBO J. (2024) 43:5469–93. doi: 10.1038/s44318-024-00236-9 PMC11574294 39333773

[8] Oh T Kim G Baek SH Woo Y Koo B-S Hwang E-H. Spatial transcriptome atlas reveals pulmonary microstructure-specific COVID-19 gene signatures in cynomolgus macaques. Commun Biol. (2023) 6:1–11. doi: 10.1038/s42003-023-05253-8 37640792 PMC10462721

[9] Kim TH Zhou H. Functional analysis of chicken IRF7 in response to dsRNA analog poly(I:C) by integrating overexpression and knockdown. PloS One. (2015) 10:e0133450. doi: 10.1371/journal.pone.0133450 26186542 PMC4505898

[10] Rise ML Hall JR Rise M Hori TS Browne MJ Gamperl AK. Impact of asymptomatic nodavirus carrier state and intraperitoneal viral mimic injection on brain transcript expression in Atlantic cod (Gadus morhua). Physiol Genomics. (2010) 42:266–80. doi: 10.1152/physiolgenomics.00168.2009 20442246

[11] Tso C-H Lu M-W. Transcriptome profiling analysis of grouper during nervous necrosis virus persistent infection. Fish Shellfish Immunol. (2018) 76:224–32. doi: 10.1016/j.fsi.2018.03.009 29510256

[12] van Muilekom DR Mueller J Lindemeyer J Schultheiß T Maser E Seibel H. Salinity change evokes stress and immune responses in Atlantic salmon with microalgae showing limited potential for dietary mitigation. Front Physiol. (2024) 15:1338858. doi: 10.3389/fphys.2024.1338858 38410809 PMC10894964

[13] Zhao X Yu H Kong L Li Q. Transcriptomic responses to salinity stress in the pacific oyster crassostrea gigas. PloS One. (2012) 7:e46244. doi: 10.1371/journal.pone.0046244 23029449 PMC3459877

[14] Qin S Xu W Wang C Jiang S Dai W Yang Y. Analyzing master regulators and scRNA-seq of COVID-19 patients reveals an underlying anti-SARS-CoV-2 mechanism of ZNF proteins. Brief Bioinform. (2021) 22:bbab118. doi: 10.1093/bib/bbab118 33907801 PMC8135462

[15] Blasi G Bortoletto E Gasparotto M Filippini F Bai C-M Rosani U. A glimpse on metazoan ZNFX1 helicases, ancient players of antiviral innate immunity. Fish Shellfish Immunol. (2022) 121:456–66. doi: 10.1016/j.fsi.2022.01.019 35063603

[16] Seaby EG Thomas NS Webb A Brittain H Taylor Tavares AL Ambrose JC. Targeting *de novo* loss-of-function variants in constrained disease genes improves diagnostic rates in the 100,000 Genomes Project. Hum Genet. (2023) 142:351–62. doi: 10.1007/s00439-022-02509-x PMC9950176 36477409

[17] Song Z Krishna S Thanos D Strominger JL Ono SJ. A novel cysteine-rich sequence-specific DNA-binding protein interacts with the conserved X-box motif of the human major histocompatibility complex class II genes via a repeated Cys-His domain and functions as a transcriptional repressor. J Exp Med. (1994) 180:1763–74. doi: 10.1084/jem.180.5.1763 PMC2191754 7964459

[18] Otten EG Werner E Crespillo-Casado A Boyle KB Dharamdasani V Pathe C. Ubiquitylation of lipopolysaccharide by RNF213 during bacterial infection. Nature. (2021) 594:111–6. doi: 10.1038/s41586-021-03566-4 PMC7610904 34012115

[19] Ripin N Parker R. Formation, function, and pathology of RNP granules. Cell. (2023) 186:4737–56. doi: 10.1016/j.cell.2023.09.006 PMC10617657 37890457

[20] Choi Y Um B Na Y Kim J Kim J-S Kim VN. Time-resolved profiling of RNA binding proteins throughout the mRNA life cycle. Mol Cell. (2024) 84:1764–1782.e10. doi: 10.1016/j.molcel.2024.03.012 38593806

[21] Gewin L Myers H Kiyono T Galloway DA. Identification of a novel telomerase repressor that interacts with the human papillomavirus type-16 E6/E6-AP complex. Genes Dev. (2004) 18:2269–82. doi: 10.1101/gad.1214704 PMC517520 15371341

[22] Levan J Vliet-Gregg PA Robinson KL Katzenellenbogen RA. Human papillomavirus type 16 E6 and NFX1-123 mislocalize immune signaling proteins and downregulate immune gene expression in keratinocytes. PloS One. (2017) 12:e0187514. doi: 10.1371/journal.pone.0187514 29117186 PMC5695606

[23] Andreeva L David L Rawson S Shen C Pasricha T Pelegrin P. NLRP3 cages revealed by full-length mouse NLRP3 structure control pathway activation. Cell. (2021) 184:6299–6312.e22. doi: 10.1016/j.cell.2021.11.011 34861190 PMC8763037

[24] Hochheiser IV Pilsl M Hagelueken G Moecking J Marleaux M Brinkschulte R. Structure of the NLRP3 decamer bound to the cytokine release inhibitor CRID3. Nature. (2022) 604:184–9. doi: 10.1038/s41586-022-04467-w 35114687

[25] Kesavardhana S Samir P Zheng M Malireddi RKS Karki R Sharma BR. DDX3X coordinates host defense against influenza virus by activating the NLRP3 inflammasome and type I interferon response. J Biol Chem. (2021) 296:100579. doi: 10.1016/j.jbc.2021.100579 PMC8081917 33766561

[26] Samir P Kesavardhana S Patmore DM Gingras S Malireddi RKS Karki R. DDX3X acts as a live-or-die checkpoint in stressed cells by regulating NLRP3 inflammasome. Nature. (2019) 573:590–4. doi: 10.1038/s41586-019-1551-2 PMC6980284 31511697

[27] Ryan CS Schröder M. The human DEAD-box helicase DDX3X as a regulator of mRNA translation. Front Cell Dev Biol. (2022) 10:1033684. doi: 10.3389/fcell.2022.1033684 36393867 PMC9642913

[28] Li J Hu L Liu Y Huang L Mu Y Cai X. DDX19A senses viral RNA and mediates NLRP3-dependent inflammasome activation. J Immunol. (2015) 195:5732–49. doi: 10.4049/jimmunol.1501606 26538395

[29] Mitoma H Hanabuchi S Kim T Bao M Zhang Z Sugimoto N. The DHX33 RNA helicase senses cytosolic RNA and activates the NLRP3 inflammasome. Immunity. (2013) 39:123–35. doi: 10.1016/j.immuni.2013.07.001 PMC3756931 23871209

[30] Nowak JK Dworacka M Gubaj N Dossimov A Dossimov Z Walkowiak J. Expression profiling of ileal mucosa in asthma reveals upregulation of innate immunity and genes characteristic of Paneth and goblet cells. Allergy Asthma Clin Immunol. (2021) 17:82. doi: 10.1186/s13223-021-00584-9 34332619 PMC8325823

[31] Ketting RF Cochella L. Concepts and functions of small RNA pathways in C. elegans. Curr Top Dev Biol. (2021) 144:45–89. doi: 10.1016/bs.ctdb.2020.08.002 33992161

[32] Rechavi O Lev I. Principles of transgenerational small RNA inheritance in *caenorhabditis elegans* . Curr Biol. (2017) 27:R720–30. doi: 10.1016/j.cub.2017.05.043 28743023

[33] Marnik EA Almeida MV Cipriani PG Chung G Caspani E Karaulanov E. The Caenorhabditis elegans TDRD5/7-like protein, LOTR-1, interacts with the helicase ZNFX-1 to balance epigenetic signals in the germline. PloS Genet. (2022) 18:e1010245. doi: 10.1371/journal.pgen.1010245 35657999 PMC9200344

[34] Ishidate T Ozturk AR Durning DJ Sharma R Shen E-Z Chen H. ZNFX-1 functions within perinuclear nuage to balance epigenetic signals. Mol Cell. (2018) 70:639–649.e6. doi: 10.1016/j.molcel.2018.04.009 29775580 PMC5994929

[35] Ouyang JPT Zhang WL Seydoux G. The conserved helicase ZNFX-1 memorializes silenced RNAs in perinuclear condensates. Nat Cell Biol. (2022) 24:1129–40. doi: 10.1038/s41556-022-00940-w PMC9276528 35739318

[36] Wan G Fields BD Spracklin G Shukla A Phillips CM Kennedy S. Spatiotemporal regulation of liquid-like condensates in epigenetic inheritance. Nature. (2018) 557:679–83. doi: 10.1038/s41586-018-0132-0 PMC6479227 29769721

[37] Abramson J Adler J Dunger J Evans R Green T Pritzel A. Accurate structure prediction of biomolecular interactions with AlphaFold 3. Nature. (2024) 630:493–500. doi: 10.1038/s41586-024-07487-w 38718835 PMC11168924

